# Polycomplexes of Hyaluronic Acid and Borates in a Solid State and Solution: Synthesis, Characterization and Perspectives of Application in Boron Neutron Capture Therapy

**DOI:** 10.3390/polym10020181

**Published:** 2018-02-13

**Authors:** Alexander N. Zelenetskii, Sergey Uspenskii, Alexander Zaboronok, Georgij Cherkaev, Alexander Shchegolihin, Bryan J. Mathis, Mikhail Selyanin, Tetsuya Yamamoto, Akira Matsumura

**Affiliations:** 1 R&D Center “Suisselle,” CEI3, rue Galilée 6, 1400 Yverdon-les-Bains, Switzerland; s.a.uspenskii@mail.ru (S.U.); georgij.cherkaev@gmail.com (G.C.); selyanine@martinex.ru (M.S.); 2 Institute of Synthetic Polymer Materials, Russian Academy of Sciences, Profsoyuznaya st. 70, 117393 Moscow, Russian; 3 Faculty of Medicine, University of Tsukuba, 1-1-1 Tennodai, Tsukuba 305-8575, Japan; bmathis@md.tsukuba.ac.jp (B.J.M.); a-matsumur@md.tsukuba.ac.jp (A.M.); 4 Institute for Biochemical Physics, Russian Academy of Sciences, Kosygina st. 4, 119334 Moscow, Russian; chembio@sky.chph.ras.ru; 5 Department of Neurosurgery, Graduate School of Medicine, Yokohama City University, 3-9 Fukuura, Kanazawa, Yokohama 236-0004, Japan; y_neuros@yokohama-cu.ac.jp

**Keywords:** hyaluronic acid, borates, solid state synthesis, Fourier transform infrared spectroscopy, nuclear magnetic resonance, boron neutron capture therapy

## Abstract

In this report, we propose a new polyborate fragment synthesis strategy along the whole chain of the polysaccharide hyaluronic acid (HA) to produce boron neutron capture therapy (BNCT) compounds. Under high pressure and deformatory solid-state conditions, polymolecular system formation takes place due to association of phase-specific transition components into a more or less distinct microscopic organization. Fourier transform infrared (FTIR) spectroscopy shows that HA and polyborates form a network of cyclic polychelate complexes. HA acts as a multidentate ligand using carboxylic and hydroxyl proton donor groups to link oxygen atoms in B–O–B bonds and borate-anions B–O(−): O–H···O, O–H···(−)O. With free electron pairs in heteroatoms –O(:)···B, –N(:)···B, HA can act simultaneously as an electron donor. Nuclear magnetic resonance (NMR) with ^13^C and ^1^H reveals a preserved complex interaction after both solubilizing and attenuating the HA-polyborate system. Stability of the product in water, low cost, ease of synthesis and scalability of manufacturing indicate that HA-polyborate complexes might have advantages over current chemotherapeutic approaches in creating therapeutic agents for BNCT.

## 1. Introduction

Recently, great progress has been achieved in the synthesis of boron-10-containing pharmaceutical compounds [1,2,3,4,5,6,7,8,9,10] for boron neutron capture therapy (BNCT). The synthesized compounds can deliver boron-10 to tumour tissues at a concentration of 40 µg/g, which is 3.5 times higher than in normal tissues. These concentrations allow for therapeutic impact on tumours while low background spares normal tissue. However, typical synthesis methods using polyhedral boron face an obstacle in the area of biocompatibility. To provide biocompatibility it is necessary to create a system of covalently-bonded elements in one complex molecule but polyhedral architecture is biologically xenogeneic leading to the need for natural carriers that also happen to cause cell growth and proliferation. These carriers include oligonucleotides, free amino acids, peptides, lipids, phospholipids and carbohydrates. Additionally, as direct bonding is typically impossible, active reagents such as diisocyanates, biscarbodiimides etc. are used as intermediate bridges (spacers) that may also have xenogeneic effects on cells. A typical polyhedral boron-containing synthesis cascade consists of multiple transformations of the original bioorganic substance, each with a risk of creating some unintended effect. We suggest here that creation of a supramolecular bond system of polyborates and HA based on repeated chelate complexes along the molecular chain and B–O–C bonds of alpha-diols with the borate is an efficient alternative to the covalent bonding of boron fragments with cell membrane-binding saccharides at the end of the multicomponent molecular chain.

**Figure 1:**
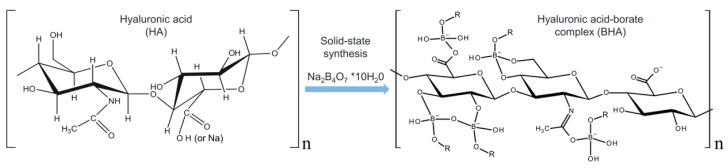
The scheme of synthesis and structure of the hyaluronic acid-borate complex. Several possible ways of coordination in the complex are presented. The interaction of the polysaccharide with Na_2_B_4_O_7_ (Borax) can lead to the formation of coordination bonds of the boron atom with all suitable electron-donating atoms. As a result, a number of complexes are formed in an aqueous solution at thermodynamic equilibrium. The totality of these complexes can be considered as a supramolecular (dynamic) system—the polysaccharide/borate/polyborate.

Thus, the aim of the study was to create a polycomplex system consisting of a polysaccharide (HA) with the most biochemically adaptive and aqueous-stable structure and a polyborax chain formed from partial tetraborate neutralization (Figure 1). The current study is also the initial step in using the proposed technology for the synthesis of complex boron and high-Z element compounds for in-sample dosimetry, tumour localization and boron dose evaluation during BNCT.

## 2. Materials and Methods

BHA solid state synthesis was conducted as follows: sodium hyaluronate of bacterial origin with a molecular weight of 1 × 10^6^ Da (Shiseido Co., Ltd., Tokyo, Japan) and sodium tetraborate decahydrate (Na_2_B_4_O_7_·10H_2_O, Sigma-Aldrich, St. Louis, MO, USA) were mixed at mole ratios of 4:1 and 1:1 and ground in an agate mortar. For reactive mixing, the two components were processed in Bridgman anvils with the deformation at a pressure of 1 GPa and a rotation angle of 500°, which is linearly related to the deformation (as described previously [11]) and at the twin-screw extruder, especially equipped for maximal forces in extrusion of solid mixtures.

In FTIR, the spectra of the tested compounds were recorded with infrared Fourier spectrometers VERTEX-70 (Bruker Optik GmbH, Ettlingen, Germany) and FTIR-1720 (PerkinElmer, Inc., Waltham, MA, USA). Depending on the physical condition of the analysed sample, appropriate spectrum recording modes such as transmission, incomplete internal reflection (IIR) and diffuse reflection-transmission (DRAFT) were selected.

NMR spectra were recorded with a Bruker Avance II 300 spectrometer (Bruker, Ettlingen, Germany) with a working frequency of 300.13 MHz for ^1^H, 75.47 MHz for ^13^C and 96.29 MHz for ^11^В in deuterium oxide (D_2_O) solutions. The concentration of solutions was 10% by mass. Dimethylsulfoxide (DMSO) (δ = 2.5 ppm) was used as an internal standard for ^1^H and ^13^C spectra and trifluoride etherate was an external standard for ^11^B (δ = 0 ppm).

In the interpretation of the HA spectra we used our own data, ASTM standards and numerous literature data [12,13,14,15].

## 3. Results

### 3.1. Fourier Transform Infrared Spectroscopy

In comparative IR spectra of the Bridgman anvil deformation products, we can observe significant differences in the spectra of the products compared to the reagents. To analyse the changes and, if possible, to determine the character of reagent reactions, the spectra are divided into regions corresponding to known, characteristic (most clearly interpretable) absorption by HA and borate groups. These regions are 3650–2750, 1750–1200 cm^−1^ (with two variants of normalization: by the C–H absorption in the valence region and deformational oscillations of these groups and also by the maximum intensive band in this spectral region), 1200–880 and 900–580 cm^−1^. Although the data are too complex to discover every facet of the reaction, primary changes in characteristic regions of the spectra in the post-reaction mixture gives a clear path to forming a hypothesis of the interaction. HA and polyborates can form a net of cyclical polychelate complexes, where HA plays the role of a polydentate ligand. It uses carboxyl and carbonyl groups as proton donors in bonds with O-atoms of B–O–B bonds and borate-anions В–О(−): О–Н···О, О–Н···(−)О and at the same time acts as an electron-donor due to the free electron pairs in heteroatoms: –О(:)···В, –N(:)···B. At the same time, the polysaccharide and polyborate form auto-associative arrangements which are typical for these compounds: intra- and intermolecular equatorial hydrogen bonds in HA in combination with dispersion interactions of cyclic pyranose rings in the axial direction. This preserves the spiral polysaccharide structure in slightly changed form and all coordinational bonds of polyborate and borate compounds with 3rd and 4th order- coordinating B atoms. This very mobile system of linkages and bonds are destroyed in accordance with the equilibrium constants of each individual complex in the given environment. Chelate formations stabilize the formation as they create a system of proton transfer to the rings to affect an anchimeric increase in the interaction.

#### 3.1.1. The Absorption Region of ОН–, NH– and СН– Valent Oscillations is 3650–2750 cm^−1^

**Figure 2:**
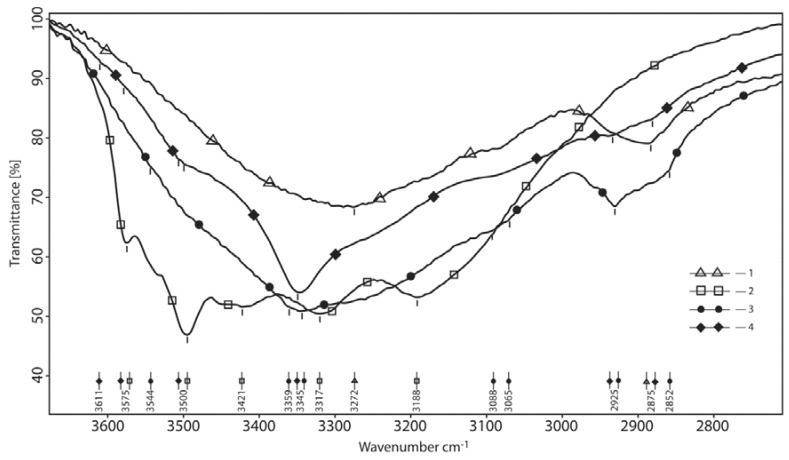
The area of С–Н, N–Н valent HA oscillations and borate oscillations (normalized by СН). 1—hyaluronic acid; 2—reaction product at the mole-link ratio of HA/g(gram)-atom B 1/4; 3—borax; 4—reaction product at the mole-link ratio of HA/g(gram)-atom B 1/1.

In the product spectra in Figure 2, redistribution by intensity and the maximum absorption bands is seen, characterizing valence vibrations of polyassociates of X–H groups compared to initial substances. Here, the peak entirely disappears at 3494 cm^−1^ of borax (the absorption by νОН– groups of crystallization water) and the peak maximum becomes more intense and narrow, located at 3350 cm^−1^. A noticeably sharp absorption maximum of OH and NH amide groups can be seen, hyperchromically shifted compared to the spectrum of HA. Such a shift is possible due to the neutralization of some COOH– groups. At the same time, the hydrogen bond structure is characterized by a greater polydispersity of associates. If we accept that there is much less water in the product than in borax, then a part of the HA hydroxyl groups and acidic borates absorb in the region close to the 3000–2900 cm^−1^. The spectrum is clearly not additive making it evident that it is slightly expanded by increasing the amount of borax. It is therefore possible that hydrogen bonds are redistributed in polycomplexes when ratios of the reagents are changed. It can be stated that the system of hydrogen bonds in HA after deformation with borax has undergone significant changes. This is particularly obvious when comparing the absorption by bound OH– groups with absorption bands of ν–CH in which the high ratio of borax/HA (green curve) in the product is overlapped by the shift of ν–ОН in the polyassociate. Thus, a particularly strong hydrogen bonding between the components of the system is shown and this fact points to the chelate structure of the binding. 

#### 3.1.2. The Area of 1750–1200 cm^−1^ of the Carbonyls and Amide Bonds 

**Figure 3:**
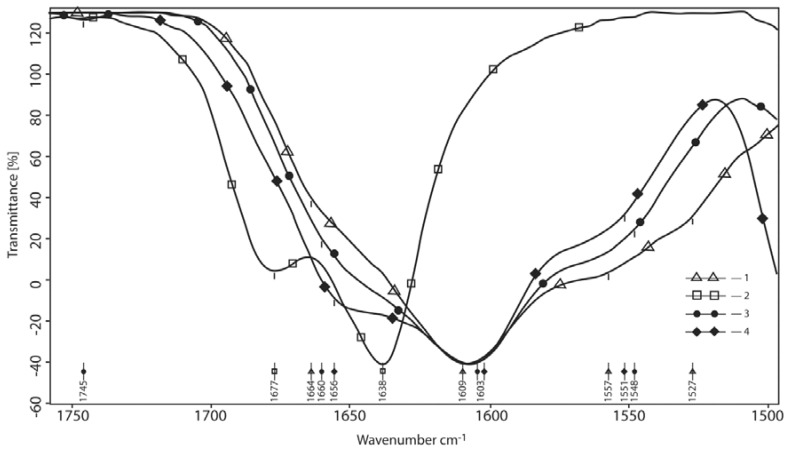
The area of carbonyls and amide bonds (normalized by the maximum intensity). 1—hyaluronic acid; 2—reaction product at the mole-link ratio of HA/g(gram)-atom B 1/4; 3—initial component—borax; 4—reaction product at the mole-link ratio of HA/g(gram)-atom B 1/1.

The following changes, which are not relevant to the additive range, can be seen in this area when normalized by the maximum intensity (Figure 3), which allows accurate determination of the change in the position of the absorption bands. Two peaks at 1677 and 1638 cm^−1^ in the borax spectrum turn into a shoulder at 1666 cm^−1^ of the main Amide-I band with an absorption maximum of 1603–1609 cm^−1^. The lower frequency shoulder at 1557 cm^−1^ (Amide-II band, with the overlapping carboxylate peak ν_a_ (CO_2_–)) slightly shifts into the low-frequency area (10 cm^−1^) allowing for accurate determination of the change in the position of the absorption bands.

**Figure 4:**
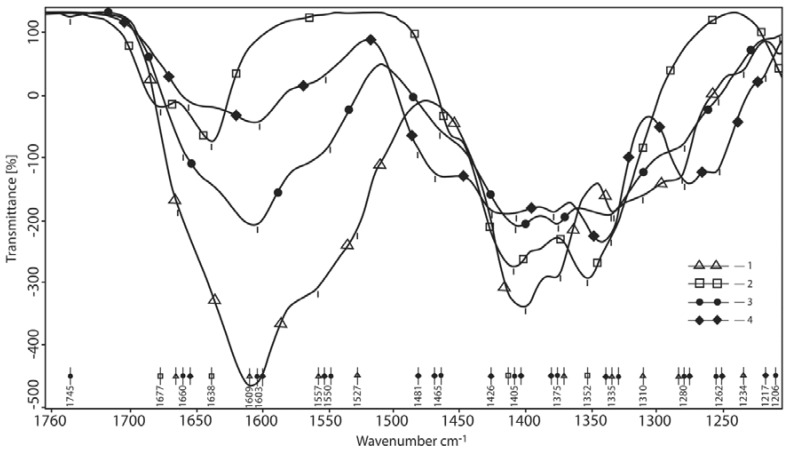
The area of carbonyls and amide bonds (the spectra are normalized by СН). 1—hyaluronic acid; 2—reaction product at the mole-link ratio of HA/g(gram)-atom B 1/4 (actually the spectrum of HA); 3—borax; 4—reaction product at the mole-link ratio of HA/g(gram)-atom B 1/1.

An alternate method is to normalize signal with respect to the fixed –CH band in the HA spectrum. (Figure 4). Using this method reveals that the absorption intensity of the Amide-I band (maximum at 1603–1609 cm^−1^) sharply descends in comparison to the HA spectrum but the shoulders of the 1660 cm^−1^ bands greatly increase in intensity. Since these bands are also likely to be Amide-I bands, it is obvious that the condition of the HA amide and carboxyl groups in the reaction product is greatly changed. In fact, they are similar to the spectra of the salt form of the acid (HA). In the acid form, HA carbonyls absorb at lower frequencies than the salt due to the hydrogen bonding of C=O···HOOC– which reduces the force constant of the bond. Such strong shifts of the carbonyl band are not new to amides and thus, in dimethylformamide, according to the degree of the association (tetramer, trimer, dimer and monomer), the Amide-I band is shifted by 90 cm^−1^: 1650, 1655, 1659 and 1741 cm^−1^ [15]. It is known that doublet of the bands 1663 and 1626 cm^−1^ in the spectrum of alpha-chitin (which is close in the structure to the HA polysaccharide with the same acetamido group) refers to νC=O oscillations, corresponding to two types of H[hydrogen]-bonds in which the C=O– group in the chitin takes part [16]. Consistent with this hypothesis is also the shift of the shoulder of the Amide-II HA band to the lower frequency region. These bands react to the destruction of associated bonds in the opposite manner of Amide-I. Similarly, dimerized formamide absorbs at 1552 cm^−1^ and the monomer at 1636 cm^−1^. The bands of the asymmetric stretching vibrations of COO(−) are in the same region, 1560 cm^−1^ but the lowered absorption frequency of the Amide-II band is evident. Peaks corresponding to the absorbance of sodium borate at 1677 and 1638 cm^−1^ disappeared in the product. Therefore, it is clear that this borate crystallization water was replaced by another ligand. The new absorption peak at 1481 cm^−1^ appears in the products, which is seen from the spectra that are normalized with respect to maximum intensity. This peak is absent from both initial materials and, therefore, must be the product of the reaction. In borate chemistry, such absorption is associated with changing of the number of B–O– bonds in the borate grid with the addition of electron-acceptor and electron-donor reagents. That is to say, it is specifically the interaction of the borate with HA by carboxyl and carbonyl groups as proton donors with O atoms of B–O–B bonds and borate anions B–O(−): O–H···O, OH···(−) O as well as the ability of free electron pairs of hetero-atoms to be electron-donors in this manner: –O(:)···In, –N(:)···B. It is significant that this peak is reduced and bathochromically shifted out (1461 cm^−1^ shoulder) as HA content in the product increases. This specific spectral region, however, is the absorption region of the B–O esoteric bonding of the boric acid, R–O–B, which is consistent with the binding principle of pyranose α-hydroxyl groups with boron (i.e., borate may be singly bonded or simultaneously bonded with two α-diol groups) [17]. The ν(CO) + δ(OH) bands, corresponding to the frequency overlapping of the valent vibrations of the C–O and deformation vibrations of OH in the HA spectrum, lie at 1399 cm^−1^ and in products #2 and #4 at 1407 and 1426 cm^−1^. Thus, aliphatic hydroxyls are strongly bonded in the product and clear borate binding can be seen. This absorption is attributed to the vibrations of C–O–B bonds in the boric acid esters. A typical borate peak appears in product #2 at 1333 cm^−1^ (borax is at 1362 cm^−1^) and this is the absorption by the В–О(−) bond, linked to the long chain of the borate bonds. In addition, according to the literature [18], the peaks at 1340 cm^−1^ correspond to asymmetrical valence oscillations (ν_a_B–O) of various borate cycles (see Figure 2). It can therefore be seen that in the product all the absorption bands vary greatly due to both the interaction with the polysaccharide and acid neutralization by the borax because, when neutralized in stages, borax forms polyborates. In equimolar acid amounts (sample #4), the borax band shows insignificant shifts. This may be due to the lower share of the bonds in C–O–B, the absorption of which overlaps the polyborate absorption bands. The two peaks which appear at 1260 and 1280 cm^−1^ in the spectrum of product #1 are bands of the ν(CO) + δ(OH) type but the bonds in the product are of a very different character than in HA (shoulder at 1280 cm^−1^). Borax does not exhibit such absorption and neither do boric anhydrides or boric acid. However, such an absorption is characteristic for tri-, tetra- and pentaborate groups that may form during partial neutralization of borax by HA in the solid state.

#### 3.1.3. Oscillations Area of С–О– Containing Bonds

**Figure 5:**
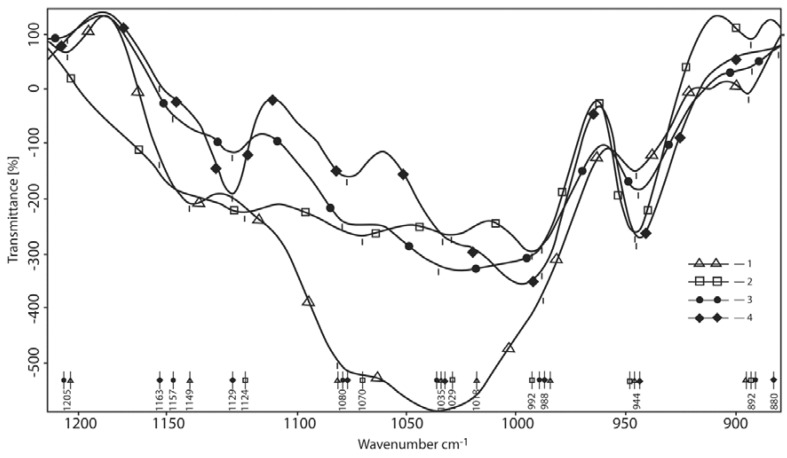
Oscillation area of the С–О– containing bonds (normalized similarly by СН). 1—hyaluronic acid; 2—reaction product at the mole-link ratio of HA/g(gram)-atom B 1/4 (it is actually the spectrum of HA); 3—initial component—borax; 4—reaction product at the mole-link ratio of HA/g(gram)-atom B 1/1.

This area contains the valence oscillations of all acetal bonds (the ring and inter-ring bridges) as well as C–O (H)–bonds of carbinols. In this region, C–O(B) bond (alkyl boric esters bond) absorption can also be assumed with high probability. The region of 1200–800 cm^−1^ is also an area of borate (BO_4_ and borate rings) valence oscillations (Figure 5).

The spectrum is normalized to CH so the redistribution of the absorption band intensity is clearly visible in the products compared to the HA spectrum. Normalization by CH should align the intensity of the spectral bands of the HA products with their ratios in the initial acid. However, in this case, we see significant changes in the spectra of products #2 and #4 not only in the intensities but also in band positions. The changes are generally qualitative and the HA spectrum is visible, albeit significantly changed. The main and the most intense signal in this region, namely the complex of the pyranose ring group bands (including their carbinol groups with the maximum at 1035 cm^−1^ and the shoulder at 1082 cm^−1^) is converted into a similar group of bands with the maximum at 995 cm ^−1^ and the shoulder at 1033 cm^−1^. Products with more HA show more similarity in bands with the HA spectrum as expected. This is not only the shift of the HA group oscillations but a complex composition of reaction band product absorption bands and the HA spectrum with the peaks redistributed. Thus, in product #2′s HA spectrum, the shoulder at 1082 cm^−1^ corresponds with a peak maximum at 1077 cm^−1^ and instead of the maximum peak at 1149 cm^−1^ the peak shifts to 1129 cm^−1^. It should be taken into account that this is an area of pendulum oscillations of the average intensity of the ammonium ion (NH^3+^). 

Ammonium may be present in partially deacetylated HA and may form a complex with boron in the final product of the type H_2_N(+)–B(−). Borax gives the absorption maxima for borates, which in these spectral regions closely coincide with the absorption maxima of the products but the ratio of these peak intensities is quite different. Finally, the common peak for all reagents is at 944–945 cm^−1^. It is intense in the spectrum of borax and less intense in the HA spectrum, reflecting their differing natures. In borates (boron esters), these are the oscillations of cyclic diborate rings while in HA they are the ν(CC) oscillations of the pyranose ring. These groups cannot interact and their absorption is added together. Thus, we observe a strong interaction of all acetal and hydroxyl (alcohol) groups of the polysaccharide with the O atoms of B–O–B bonds and borate anions B–O(−) at the same time by means of free electron pairs and hydrogen atoms –О(:)···В, О–Н···О, О–Н···(−)О. In the cyclic framework, this translates to the formation of chelating complexes with an almost quantitative yield ratio of 1:1. Product #4′s spectra, where the HA amount is much higher, has a strong imposition of polysaccharide bands but the interaction is plainly visible. Furthermore, this absorption region of the C–O–B bonds is by means of the reaction: В–О–В + НО–С– → В–ОН + В–О–С. We clearly see this equilibrium in the NMR spectra of the products in aqueous solutions.

#### 3.1.4. Area of the Deformational and Packaging Oscillations (Overall Normalization by CH)

**Figure 6:**
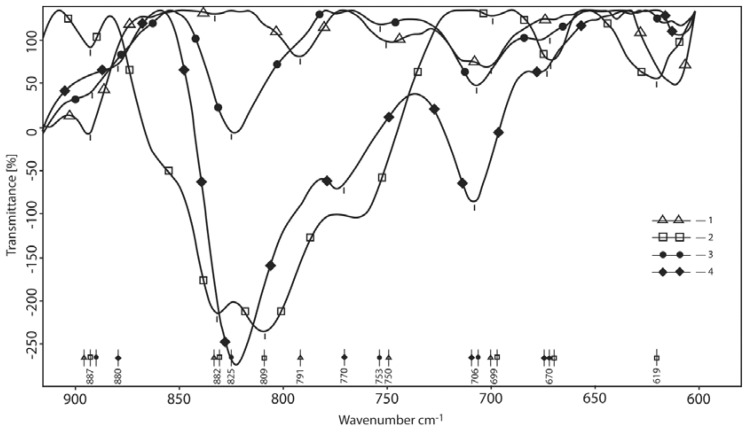
Area of the deformational and packaging oscillations (overall normalization by CH). 1—hyaluronic acid; 2—reaction product at the mole-link ratio of HA/gram-atom B 1/4; 3—initial component—borax; 4—reaction product at the mole-link ratio of HA/gram-atom B 1/1.

Changes in deformational and packaging oscillations are also significant (Figure 6). It should be noted that the normal modes of oscillations are so mixed and delocalized that it is impossible to describe the complex wave motion with a simple single term. Therefore, description will be limited to a statement of changes in the spectra of initial compounds that primarily indicate the transformation of their molecular packing and the formation of a new system of intermolecular bonds. The peaks at 882 cm^−1^ in the spectra of borax and HA disappeared in the spectra of the products. This indicates the disappearance of out-of-plane, deformational oscillations of the O–H (COOH) at 900 cm^−1^ after the reaction due to neutralization of these groups and their conversion to carboxylated forms. The product displayed a single peak at 820–825 cm^−1^. In the borax region of this spectrum, there are two absorption maxima at 808 and 831 cm^−1^. HA has a peak at 791 cm^−1^, which disappears in the products. Also, there are no peaks of borax in the spectra of products at 678 and 620 cm^−1^ but the peak of HA remains at 615 cm^−1^ from the variable oscillation of NH^+^.

### 3.2. ^1^H and ^13^C NMR Spectra of the Polycomplexes

**Figure 7:**
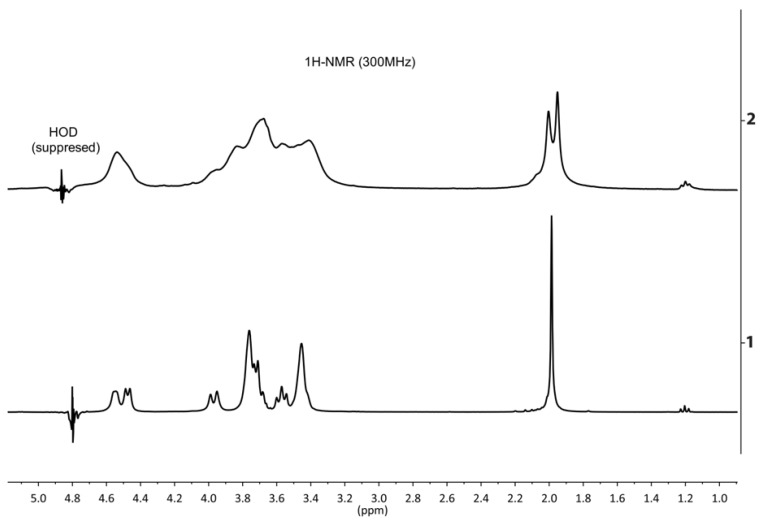
^1^H NMR spectra of the alkaline hyaluronic acid (1) and the hyaluronic acid, borax and potassium hydroxide solution in a molar ratio of 1:4:4, respectively (2). рН = 14 in all solutions. The borate formation is indicated by the general broadening of the spectrum and the splitting of the signal of the protons of the acetyl group (about 2 ppm). The triplet with the chemical shift of 1.17 ppm belongs to the methyl group of the ethanol internal standard.

The complex interaction is preserved even in solubilisation and a potent dilution of the HA-polyborate system. In the proton spectrum of an aqueous hyaluronic acid solution (1:1, mole/mole) with borax and potassium hydroxide in a molar ratio of 1:4:4, respectively, general broadening of the signals is observed (Figure 7). 

**Figure 8:**
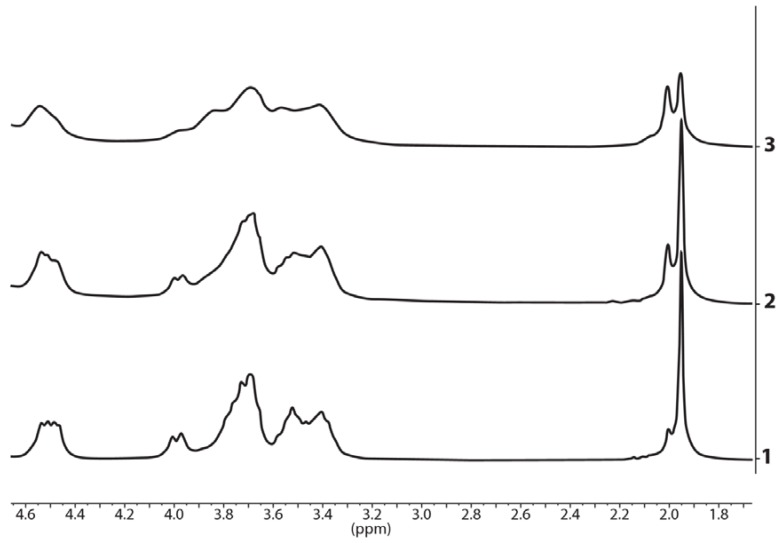
^1^H NMR spectra of the hyaluronic acid in the alkaline solution (1), the mixture of hyaluronic acid and borax in a molar ratio of 1:1 (2) and the mixture of hyaluronic acid, borax and potassium hydroxide in a molar ratio of 1:4:4 (3), respectively. рН = 14 in all solutions.

**Figure 9:**
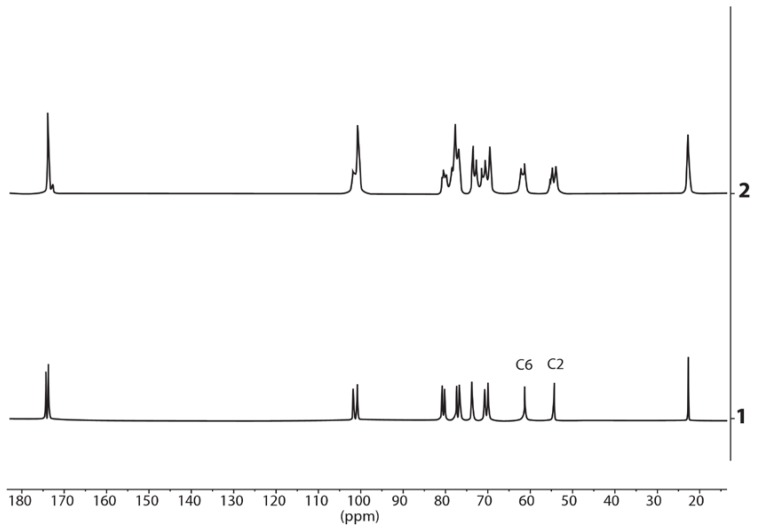
The spectra of ^13^C hyaluronic acid in alkaline solution (1) and the mixture of hyaluronic acid, borax and potassium hydroxide in a molar ratio of 1:4:4, respectively (2). рН = 14 in all solutions. The borate formation is indicated by the splitting of the signal of carbon atoms C2 (about 55 ppm) and C6 (about 62 ppm) of the *N*-acetylglucosamine moiety.

An indication of borate derivative formation is the presence of two singlet signals from the protons of the acetyl groups which differ in the chemical shift by the value of 0.05 ppm (15.6 Hz). Relative integrated intensities of these signals are dependent on the ratio of HA:borax (Figure 8). We can also estimate the formation of the borate derivatives in the above-mentioned mixtures by splitting the signals of carbon atoms C2 and C6 of the *N*-acetylglucosamine moiety in the ^13^C spectrum (Figure 9).

According to rheological studies of diluted and semi diluted solutions of such complexes [19], sodium hyaluronate chains in the presence of borax are not prone to strong crosslinking. With the addition of borax, the viscosity sensitivity to temperature decreases—the effective activation energy is reduced from 20.4 to 16 kJ/mol. Such behaviour indicates an increase in chain flexibility in the sodium hyaluronate in the presence of borax, forming polyborates. This is a result of the screening of electrostatic interactions and the impact of borax on sodium hyaluronate macromolecular conformation (and therefore on the viscosity of its solutions). The formation of complexes with borates may lead to high magnitude increases in the rigidity of the sodium hyaluronate chains and even affect cross-linking in solutions with high borate concentrations.

## 4. Discussion

Various biologically active molecules that are selectively accumulated by tumour cells can be used as transporters in the synthesis of BNCT compounds with the knowledge that modification of biomolecules by inclusion of a boron fragment can significantly influence their biological properties. This may lead to a false mode of thinking whereby as much boron as possible is crammed into the polyhedron. However, the larger the polyhedron, the more diverse the transport component might be in its chemical structure or size and therefore the boron-containing fraction might be diluted by high molecular weight carriers. Furthermore, the toxicity of such compounds needed to carry high-boron polyhedrons might influence the effective therapeutic dosage for injection. With this in mind, a change to a verified, non-toxic carrier allows for increasing boron concentration by an order of magnitude, thereby solving any problems with over dilution as one gram-mole of the compound would deliver 6 × 10^23^ molecules. It is plain to see that, for this reason, therapeutic plans must find the optimum intersection of the boron concentration and carrier size/toxicity curves. 

Among the second-generation compounds used in BNCT one consists of polyhedral borane (BSH, [20]); another contains one boron atom in organoboric acid (BPA, [21,22,23]) and the efficacy of these compounds is far from perfect [24]. Another problem is the boron-10 isotope yield in the final product which depends on the boron-10-enriched original compound. Regardless of method, the synthetic production of polycyclic boron hydrides suffers from low yield of the polyhedron. Moreover, at each stage of the compound synthesis, the boron-10 isotope yield decreases and does not exceed a few percent.

In our study, selected ratios of boron gram-atom to HA mole-link were 4:1 and 1:1, respectively. The selection of ratios is dictated by the following reason: if the cyclic form of pyranose is combined with boron in a 2:1 molar ratio, 2 cyclic rings will form from the inter-ring linkage. At a 1:1 ratio, 1 cyclic ring is formed. Since HA mole-linkage consists of 2 cyclic rings, heteroborate links will form along the HA chain without deformation of the spatial grid that can cause severe changes in system viscosity. However, to precisely form this product, the task we have defined necessitates the selection of optimal boron concentrations in the mixture. From a BNCT manufacturing standpoint, it is better to saturate the product with as much boron as it can hold but for optimal boron accumulation in a tumour, strict stoichiometry of the complexes should be defined to ensure a maximal level of supramolecular binding.

It is known that claw-type complexes have a high binding energy and are very stable [25]. In conditions of high pressure and shear forces, polymolecular systems are formed by association with their transition to a specific phase having a more or less clearly defined microscopic organization. According to the definition given by Len, “Supramolecular chemistry—is a ‘chemistry beyond the molecule’ that studies the structure and functions of associations of two or more chemical species held together by intermolecular forces” [25].

Chelate complexes are most stable as they establish a system of cyclic transfers of protons and electron pairs that enhance the reaction in an anchimeric fashion. Binding of sugars with borate into an ion complex is a well-known reaction widely applied in the analytical chemistry of both boron and sugars [17,26,27,28]. As with all supramolecular systems, the polycomplex is expected to be stable in water solutions and its tumour selectivity can be increased by HA. As HA possesses multiplex interactivity with biological tissues, it is ideal as a carrier for targeting delivery of active compounds fused to it by synthetic processes [29]. It can interact with the fibroblast cell surface, as it selectively bonds to CD44 and RHAMM- specific receptors on the surface of the cytoplasmic membrane [30]. Due to its natural origin, HA shows such advantageous features as biocompatibility, biodegradability and nonimmunogenity. HA acts as a cellular signalling molecule during inflammation, wound healing, or cancer metastasis [31,32,33]. HA, together with its receptor (CD44) and degradation enzymes (HYAL-1, etc.), acts as a carcinolytic agent, suppressing tumour growth and metastasis and this is evidenced by accumulation of HA and its associated degradation enzymes within malignant tumours [29]. HA shows very fast elimination from all tissues and a long history of use in cosmetology proves its harmlessness even in very large doses [34,35]. Despite a number of studies devoted to mono- and disaccharides [17,28] as of this report we are not aware of publications about complexing polyborates with HA.

Although most drugs are manufactured in liquid form, the more efficient method in the case of HA is reactionary allegation in a solid state (RASS) at high pressure and shear stress. A liquid phase method is inefficient because HA solutions are viscous and their reagent concentration is not high meaning that solution preparation requires prohibitively large amounts of water. On the other hand, polymer deformation at a high pressure maintains higher reagent concentrations. Additionally, deformation processing leads to the formation of charged conditions in the substance mass, disordering of its structure and polarization of molecules and these processes are intensified in the mutual deformation of biocomponent polymer mixtures. In spite of the rapid acceleration of the relaxation processes after pressure release, deformation-induced structural changes are often preserved, maintaining metastable structures even under elevated intrinsic energy conditions. Polymer mixtures (with or without low molecular weight reagents) have an additional advantage in that they can be exposed to elevated pressure and shear deformation to remove diffusion limitations that exist in reactions in solid state or highly viscous media. When subjected to high pressure and shear deformation (even in an extruder), unique but reliable behaviour is observed in mixtures of highly organized compounds with high cohesive energies such as cellulose, chitin and chitosan [36]. It is therefore relatively easy to produce chitosan salts with a variety of solid organic acids in high quantitative yield using a RASS-based extrusion method [37,38]. Until the present work, the solid-phase interaction of polysaccharides and, in particular, hyaluronic acid has not been studied.

Several issues should be addressed before further preclinical evaluation of the developed compound. Regarding the components of the synthesis, borax naturally causes alkalinisation of the solution. In NMR analysis, all tested solutions were made strongly alkaline, which reduces signal broadening in NMR spectra caused by both the high viscosity of HA, which decreases with an increase in the pH of the solution [39] and the inverse change in the sp^2^/sp^3^ states of the boron atom, where at a high concentration of alkali almost all boron atoms become tetracoordinate boron atoms (turn into the sp^3^ state). For the initial phase of biological experiments, we will need to adjust the pH to physiological ranges before performing the cytotoxicity tests.

The toxicity of any new anticancer compound is an important issue to be addressed before preclinical evaluation and prospective clinical use. Despite the abovementioned stability of claw-type complexes in general and the stability of synthesized compound in the tested solutions, changes in environmental conditions in biological systems can affect the complex and might lead to liberation of the borate species. Moreover, during BNCT itself, boron is destined to decay after the neutron irradiation. In such a case, if a boron complex is present in tumour cells, the toxicity of the decay products will cause an additional therapeutic effect, though presence in healthy tissues will cause unwanted side effects of the drug. Regarding this issue, the main task before biological testing will be the verification of the stability of the preparation in biological media and cell cultures.

In oncology, adjuvant therapy methods often balance on the verge of heal and harm for the organism as a whole, trading known risk for a guaranteed therapeutic effect. As our components are at the initial stage of development, we believe that the important issue of toxicity and the expected balancing of risk and therapeutic effect will be taken into account at all stages of further experiments.

## 5. Conclusions

BNCT is a promising method over standard radiation therapy from a standpoint of efficacy and safety while being unique in its influence on invasive tumours. However, enhancing the target effect of the neutron beam has been a key focus of translational research. Here, we report that BHA is a highly water stable and easy-to-manufacture compound. A description of the synthesis was provided, purity and concentration were determined empirically through FTIR and NMR and the full analysis of the HA-polyborate complex was elaborated. At this stage of the chemical analysis we can indicate that, compared to currently utilized BNCT agents, the proposed HA-polyborate complexes are characterized by the low cost of reagents, ease of synthesis, scalability of manufacturing and stability in water. 

Of much interest in biomaterial engineering is the modification of existing compounds with additional functional groups to increase efficacy, solubility, delivery and specificity [39]. Peptides, organic groups and other inorganic elements might eventually be blended in specific ratios to give custom targeted therapy. To this end, hyaluronic acid is an excellent scaffold to attach these functional modifications to and we have shown that our HA-polyborate modification holds promise to benefit cancer therapy. Now that a cheap and easily synthesized HA compound is available, future research will be intensely focused on modification and empirical determination of the best functional groups to add to HA-polyborate complexes for customized results.
